# Laparoscopic repair and total gastrectomy for delayed traumatic diaphragmatic hernia complicated by intrathoracic gastric perforation with tension empyema: a case report

**DOI:** 10.1186/s40792-022-01477-8

**Published:** 2022-06-20

**Authors:** Mayuko Kori, Hidetoshi Endo, Kazuhiro Yamamoto, Nobuyasu Awano, Takuo Takehana

**Affiliations:** 1 Department of Gastroenterological Surgery, Saku Central Hospital Advanced Care Center, Nagano, Japan; 2 Department of Respiratory Medicine, Japanese Red Cross Medical Center, Tokyo, Japan

**Keywords:** Delayed traumatic diaphragmatic hernia, Laparoscopic repair, Intrathoracic gastric perforation, Tension empyema, Laparoscopic total gastrectomy

## Abstract

**Background:**

Blunt traumatic diaphragmatic hernia (TDH) is a complication of blunt diaphragmatic injury. If missed, it could lead to critical presentations, such as incarceration or strangulation of the herniated intra-abdominal organs, and thus, early surgical repair is required. Methods of the operative approach against delayed TDH remain unclear. Even with the spread of the minimally invasive approach, laparotomy has been predominantly selected for cases with hemodynamic or gastrointestinal complaints. Literature on the use of laparoscopy for repair of such cases is limited, and no study has been conducted for those with intrathoracic gastric perforation.

**Case presentation:**

A 55-year-old male patient with a history of multiple traumas presented with shock, followed by left hypochondrium pain and vomiting. The patient was admitted to the emergency department of our institution and diagnosed with delayed TDH complicated by intrathoracic gastric perforation, and tension empyema. Emergency surgery using laparoscopic approach was performed, despite unstable hemodynamics, considering orientation, exposure, and operativity compared with laparotomy. Repair of the diaphragm plus total gastrectomy was successfully performed by minimally invasive management. The patient made an uneventful recovery without recurrence after 8 months.

**Conclusion:**

Unstable hemodynamic conditions and intrathoracic gastric perforation could not be contraindications to laparoscopic repair in treating delayed TDH.

## Background

Blunt traumatic diaphragmatic hernia (TDH) is a complication of blunt diaphragmatic injury frequently caused by traffic accidents and accounts for approximately one-third of traumatic diaphragmatic tears [1, 2]. Because other associated injuries are often comparatively severe, blunt diaphragmatic hernia could be missed at the time of injury and may lead to delayed presentations, including visceral obstruction, strangulation, and perforation, which are critical complications [3].

Surgical management for TDH can be classified into four approaches: transabdominal by either laparotomy or laparoscopy and transthoracic by either thoracotomy or thoracoscopy. Owing to the high incidence of intra-abdominal organ injuries, the transabdominal approach is dictated for exploration and repair [4]. Although no surgical management for isolated delayed TDH has been recommended, some recent studies have implied the efficacy of laparoscopic approach of repair [5–7]. However, in most cases of strangulation and necrosis in the herniate viscera, treatments highly relied on laparotomy (and additional thoracotomy if necessary) due to indication for transabdominal exploration and manipulation [4, 5]. Few studies have been conducted on a successful laparoscopic approach in cases complicated by intestinal strangulation. For cases with gastric perforation, none have selected laparoscopic approach for repair.

Here, we report the case of a patient diagnosed with delayed TDH accompanied by intrathoracic gastric perforation and tension gastrothorax, and diaphragmatic repair with total gastrectomy was performed using the laparoscopic approach.

## Case presentation

A 55-year-old male patient was involved in an accident during work 8 months ago and was hit by a fallen tree and had spinal cord injury, vertebral fracture (Th10/11), fracture of the spinous process (Th8–L1), multiple rib fracture, right hemopneumothorax, and left pneumothorax. The patient was admitted to the emergency department and intensive care unit (ICU) of our institution. His clinical course during the acute phase was remarkable for an episode of emergency resuscitative thoracotomy (which was unremarkable for any diaphragmatic injuries), resuscitative endovascular balloon occlusion of the aorta, acute renal injury treated by temporary blood purification therapy, and multiple deep venous thromboses requiring anticoagulation. The patient was transferred to a rehabilitation hospital after 2 months. Paraplegia of the lumber region and lower limbs due to spinal cord injury remained; however, the patient could get on a wheel chair without assistance. His regular medication included apixaban.

During the patient’s stay in the rehabilitation hospital, he presented with left hypochondrial pain and vomiting after training. The symptoms persisted for 3 days, and the patient had recurrent coffee ground emesis accompanied by blood pressure reduction. A proton pump inhibitor was administered. The patient was diagnosed with shock due to possible bowel obstruction at the previous rehabilitation hospital and was thus immediately transferred to the emergency department of our hospital. On arrival to our emergency department, vital signs were remarkable for a fever of 39.3 °C, heart rate of 132 bpm, respiratory rate of 40 bpm, blood pressure of 87/51 mmHg, and oxygen saturation of 98% with 3 L/min oxygen delivered via nasal cannula. Physical examination revealed decreased breath sounds in the left lower lung fields. On inspection, thoracic expansion was decreased in the left thorax. The patient complained of spontaneous pain and tenderness of the left hypochondrium. Laboratory tests showed elevated inflammatory markers, that is, C-reactive protein (30.3 mg/dL) and leukocyte count (23,500/μL), decreased renal function (i.e., serum creatinine of 1.24 mg/dL), and increased total bilirubin (i.e., 1.9 mg/dL). Arterial blood gas analysis (3-L/min oxygen via nasal cannula) showed PO_2_ of 87Torr, PCO_2_ of 30 Torr, pH of 7.51, alveolar arterial difference of oxygen (A-aDO _2_) of 126 Torr, and lactate of 2.44 mmol/L.

**Fig. 1:**
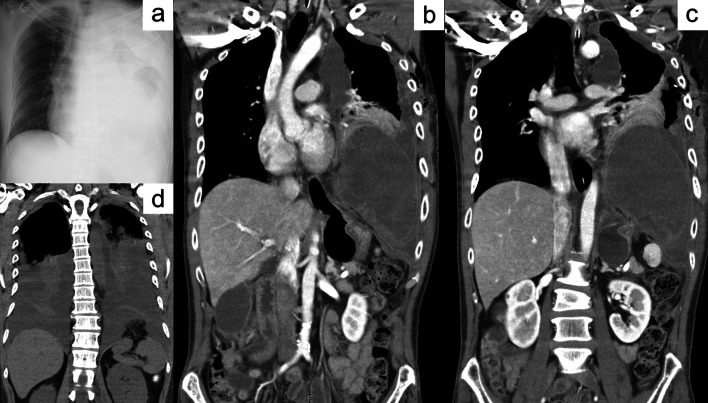
Preoperative imaging findings. **a** Chest X-ray showing the opacity of the entire left lung field with pleural fluid and left intrathoracic free air. The trachea and mediastinum shifted to the right. **b** Coronal images of the enhanced computed tomography (CT) scan on admission revealed that the fundus and corpus of the stomach with reduced contrast enhancement herniated to the left thoracic cavity through a defect in the diaphragm. The cardia and pylorus of the stomach presented normal enhancement. **c** The left lung is excluded by the gastric content of the herniated body, intrathoracic free air, and pleural effusion. **d** A plain CT scan obtained during the patient’s previous admission showed a small defect in the left hemidiaphragm with a herniated omentum

Chest X-ray was remarkable for the opacity of the entire left lung field with pleural effusion, and the trachea and mediastinum shifted to the right side (Fig. 1a). A thoracostomy tube was inserted into the left fourth intercostal region for drainage. Approximately 470-mL brownish dark red, turbid, malodorous pleural fluid was removed via the catheter. *Streptococcus mitis* was detected from the culture of the pleural flood. Contrast-enhanced thoracoabdominal computed tomography (CT) revealed that the fundus and corpus of the stomach herniated through the left hemidiaphragm, collapsing the left lower and upper lobes of the lung. The herniated gastric wall showed reduced contrast enhancement with intramural gas as evidence of necrosis. Intrathoracic free air and pleural effusion suggested gastric perforation. The cardia and pylorus of the stomach appeared intact, showing normal enhancement (Fig. 1b, c). The nasogastric tube had no effect on gastric decompression because it could not reach the prolapsed fundus.

Retrospectively, follow-up CT performed 7 months ago during the previous admission (i.e., shortly before the patient was discharged from our institution) was remarkable for TDH with omental prolapse, which appeared to have been overlooked (Fig. 1d). The aforementioned findings have led to the conclusion that the patient had delayed TDH that enlarged over time and caused incarceration and strangulation of the stomach. The patient’s condition was aggravated by intrathoracic gastric perforation, and tension empyema, leading to septic and obstructive shock. Fluid resuscitation and antibiotic therapies were immediately administered.

**Fig. 2:**
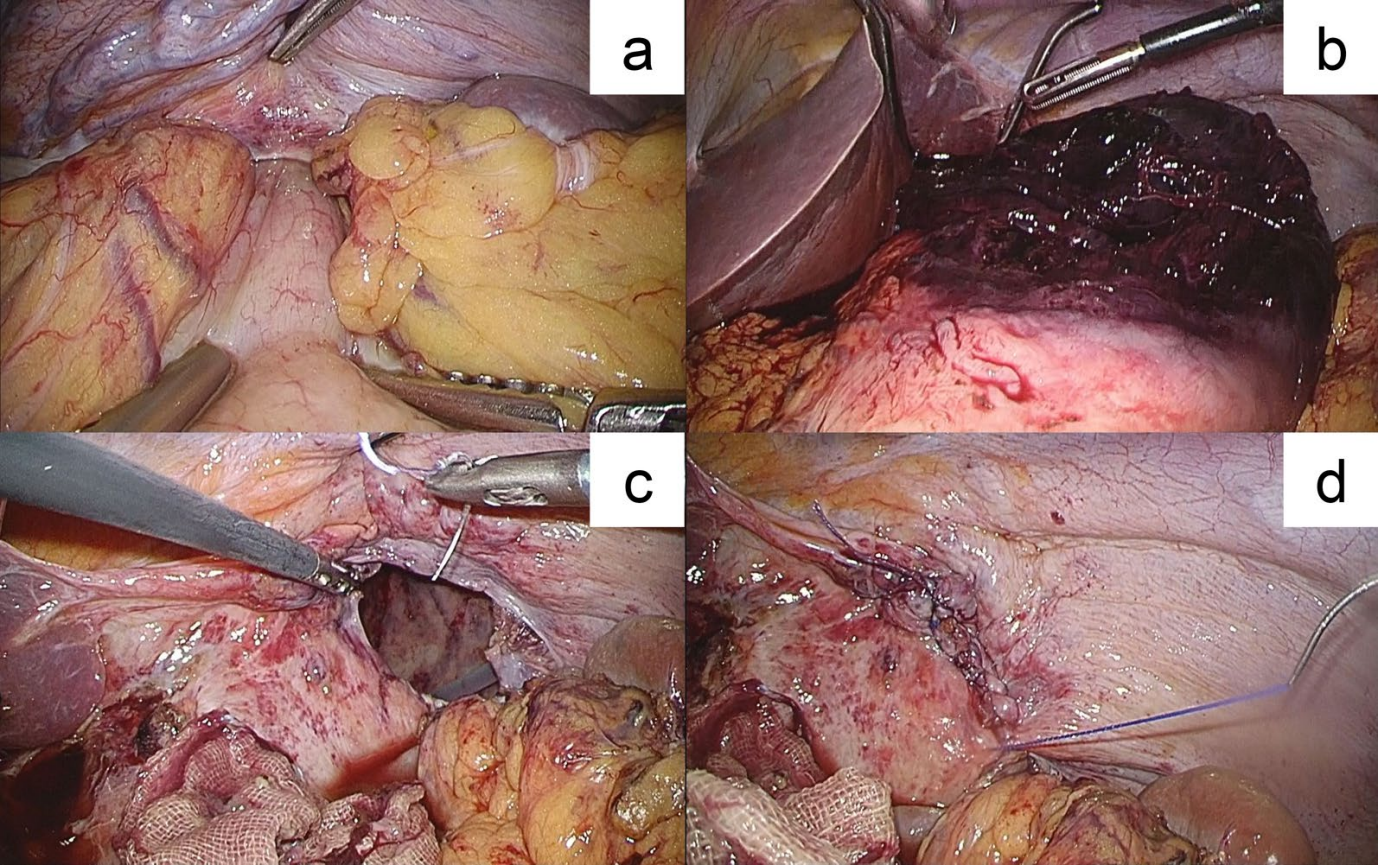
Surgical findings. **a** Precise exploration of the abdominal cavity and diaphragm revealed that the stomach incarcerated into the left thoracic cavity through a hernial defect in the left hemidiaphragm. The intraperitoneal body of the stomach was viable. **b** The herniated body was pulled back to the abdominal cavity after hernia orifice enlargement and gastric content aspiration. The fundus and corpus of the stomach were necrotic. **c** The hernia defect before closure was estimated to be 4 cm in the major axis after incision. The thoracic cavity could be observed and were drained through the defect. **d** The hernial defect was completely closed using a nonabsorbable running suture

**Fig. 3:**
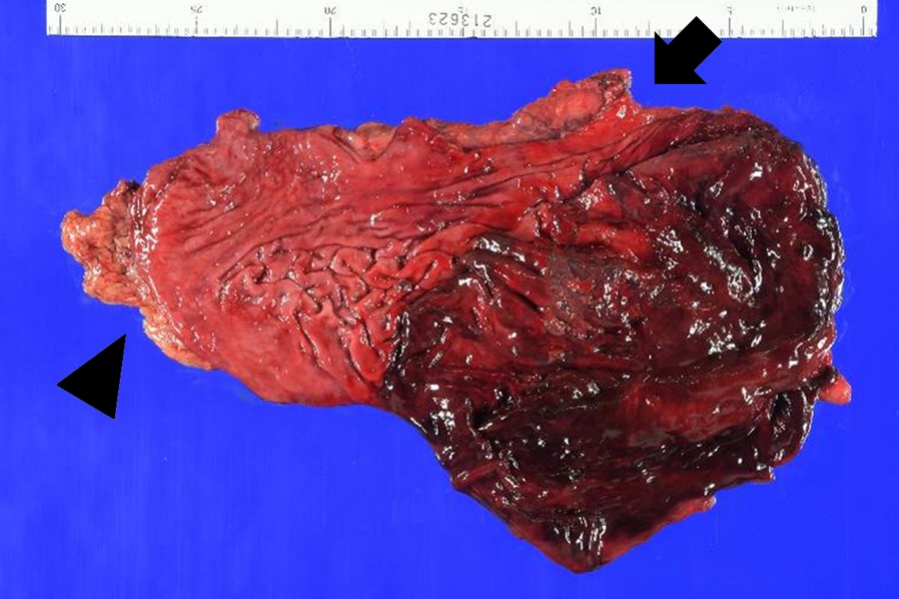
Postoperative finding. Postoperative observation of the resected stomach was remarkable for necrotic and perforated fundus and corpus. Although the necrotic area extended to the vicinity of the cardia, the cardia (arrow) and pylorus (arrowhead) were intact

Surgical consultation was performed, and we decided that urgent pleural lavage, drainage of the necrotic tissue, reduction of the hernia content, and repair of the diaphragmatic defect were necessary. Emergency surgery was performed on the same day. Although the patient’s hemodynamics were improved, it were not fully stabilized. However, the laparoscopic approach was intentionally selected to obtain a better surgical view and operativity of the diaphragm and thoracic cavity, with the preparation for immediate conversion to laparotomy, thoracotomy (if necessary), or thoracoscopy. Laparoscopy allowed a thorough observation of the abdominal cavity and diaphragm, which showed that the stomach herniated to the thoracic cavity through a diaphragmatic defect in the medial area of the left hemidiaphragm (Fig. 2a). No ascites or contamination in the peritoneal cavity was observed. Other intra-abdominal organ injuries were absent. We enlarged the hernia orifice by ventral incision (estimated to be 4 cm with a diameter at the longest); however, reducing back the incarceration remained difficult due to dilation of the dislocated stomach. Thus, the gastric content was aspirated, which enabled us to gently pull back the stomach to the abdominal cavity. Severe ischemic necrosis was observed from the fundus to the corpus of the stomach, reaching just below the cardia (Fig. 2b). It was concluded that the stomach could not be conserved, and total gastrectomy must be performed. After the necrotic area was excised, we inspected the left thoracic cavity through the widened hernia orifice. The left pleural cavity was contaminated with gastric content and necrotic tissue. Additional three ports were inserted into the left thoracic cavity to support lavage and drainage. The thoracic tube inserted preoperatively was confirmed to have been placed dorsally as intended. Surgical debridement was performed as far as possible with attention not to damage the lung parenchyma. The hernia defect was closed using a nonabsorbable running suture, and Roux-Y anastomosis was used for reconstruction (Fig. 2c). The surgery was completed laparoscopically by draining the left subdiaphragmatic space and the dorsal side of the anastomosis. Vital signs under anesthetic management remained stable throughout the surgery. The operation time was 309 min, and estimated blood loss, including the gastric content and pleural effusion, was 980 mL. Blood transfusion was not administered. The fundus and corpus of the resected stomach were necrotic with multiple perforations, whereas the cardia and pylorus were intact (Figs. 2d and 3).

The patient was placed in the ICU with ventilator assistance and vasopressor circulatory support. The patient was extubated on postoperative day (POD) 1 and was discharged from the ICU on POD 2 without vasopressor circulatory support. The thoracic tube was replaced on POD 8 because follow-up CT revealed residual empyema above the left hemidiaphragm, for which the formal tube seemed ineffective. Antibiotic treatment was continued until POD 9, and the thoracic tube was removed on POD 10. CT performed on POD 15 showed reduced abscess. Chest X-ray showed gradually decreasing pleural fluid, and laboratory data demonstrated declined inflammatory markers. No signs of surgical site infection were observed throughout the postoperative course.

Due to even worsened performance of activities of daily living (ADLs), the patient required additional social support before discharge and was transferred back to the rehabilitation hospital on POD 39. Since then, the patient has been asymptomatic without any signs of recurrence until his latest visit 8 months after the operation.

## Discussion

In this report, we described the case of a patient with delayed TDH complicated by intrathoracic gastric perforation, leading to tension empyema, which was surgically treated with a complete laparoscopic approach, despite unstable vital signs. To the best of our knowledge, this is the first case to be treated with endoscopic surgery.

Blunt TDH is caused by indirect force that suddenly increases the intra-abdominal pressure, leading to diaphragmatic tears [2, 3]. While most blunt diaphragmatic injuries are due to motor vehicle accidents, some are induced by fall and collision traumas, as was in the present case [8, 9]. The evaluation and diagnosis of blunt TDH could be missed or delayed for two reasons. First, it is often accompanied by associated injuries (e.g., cardiovascular system, liver, spleen, kidney, and lung), which are relatively severe. Second, it may initially present no symptoms, particularly when the defect is small [2, 8, 10]. Since the defect cannot be closed spontaneously because of the continuous negative pressure of the thoracic cavity and movement of the diaphragm, it can cause visceral hernias [2, 3]. If blunt TDH cases are missed, mortality due to late-onset complications is as high as 30%, and when the prolapsed organ is strangulated, this rate can reach 66% [10–12]. Therefore, early surgical intervention is essential when a diagnosis is made.

There are four surgical approaches against TDH with a combination of transabdominal/transthoracic and open incision/endoscopic operation. Recently, with the spread of endoscopic surgical approach, laparoscopic and thoracoscopic approaches against TDH have been selected for patients with stable systemic condition or those without associated organ injuries [2, 4–7, 13–19]. Minimally invasive management is superior to open procedures in terms of postoperative pain, time of recovery, and risk of surgical site infections [12, 16–20]. A retrospective study has indicated decreased hospital stay in patients with laparoscopically repaired, isolated, diaphragmatic injuries compared with that in patients who underwent laparotomy [16]. In contrast, disadvantages are indicated in cases with major hemorrhage or uncontrollable shock, where laparotomy/thoracotomy is usually recommended [12, 16].

Because delayed TDH is uncommon, the optimal surgical approach has not been proposed. Both transabdominal and transthoracic approaches could be used; however, in cases with ischemic or gangrenous herniated viscera, the abdominal approach is usually recommended [2, 5, 21]. In severe cases with unstable vital signs or associated organ injuries, repair of delayed TDH has traditionally relied on transabdominal or thoracoabdominal approaches [22]. However, more surgeons are selecting laparoscopy as an initial approach to delayed TDH complicated by associated organ injuries, for example, bowel strangulation, most of which were successfully repaired by minimally invasive surgery without conversion to laparotomy/thoracotomy [13–15, 23]. We expect that the range of indications of laparoscopic surgery would expand based on surgeons’ sufficient expertise and skills.

Our institution has aimed to select minimally invasive approaches over both elective and emergency surgery and has shown the efficacy of laparoscopic Hartmann’s procedure for diffuse peritonitis caused by perforation in the left-sided colon [24]. Despite different pathological conditions, we suggest that provided with the surgeons’ sufficient experience and techniques, laparoscopic approach could be safe and effective even in cases with severe general conditions. In the present case, laparoscopic surgery was chosen over laparotomy despite unstable hemodynamics for two reasons: (i) it provides a better visual field leading to a precise and detailed observation of the diaphragm, and (ii) the operativity of laparoscopic forceps was beneficial for approaching the diaphragm and the thoracic cavity toward the cranial direction. Additionally, we anticipated that respiratory distress and occlusive shock due to tension gastrothorax would be relieved by reducing the incarcerated stomach. As a result, the patient could tolerate the operation, and the operative duration was assumed appropriate considering the time required for total gastrectomy plus reconstruction and thoracic cavity drainage. The patient’s postoperative pain was difficult to assess owing to the previous injury and paraplegia. Time of postoperative recovery and hospital stay were prolonged due to declined ADL performance that required strong support. Nevertheless, no surgical site infections were detected during the postoperative course.

## Conclusion

We reported the case of a patient with delayed TDH complicated by intrathoracic gastric perforation that led to tension empyema, which caused septic and obstructive shock. Considering its advantages in surgical exposure, orientation, and operability, the laparoscopic surgery was selected over laparotomy and the surgery was completed safely. Our experience demonstrates that laparoscopic repair of delayed TDH could be a safe and effective surgical method, even in hemodynamically complicated situations, provided that surgeons have abundant experience and meticulous technique.

## Data Availability

Data sharing is unavailable due to patient privacy concerns but are considerable from the corresponding author on reasonable request.

## Abbreviations

TDH: Traumatic diaphragmatic hernia
ICU: Intensive care unit
CT: Computed tomography
POD: Postoperative day
ADL: Activity of daily living
